# Ambient air pollutants and breast cancer stage in Tehran, Iran

**DOI:** 10.1038/s41598-024-53038-8

**Published:** 2024-02-16

**Authors:** Zahra Khorrami, Mohsen Pourkhosravani, Ali Karamoozian, Ali Jafari-Khounigh, Mohammad Esmaeil Akbari, Maysam Rezapour, Reihaneh Khorrami, Seyed Mahmood Taghavi-Shahri, Heresh Amini, Koorosh Etemad, Narges Khanjani

**Affiliations:** 1 Ophthalmic Epidemiology Research Center, Research Institute for Ophthalmology and Vision Science, Shahid Beheshti University of Medical Sciences, Tehran, Iran; 2 Department of Geography and Urban Planning, Shahid Bahonar University of Kerman, Kerman, Iran; 3 Modeling in Health Research Center, Institute for Futures Studies in Health, Kerman University of Medical Sciences, Kerman, Iran; 4 Road Traffic Injury Research Center, Tabriz University of Medical Sciences, Tabriz, Iran; 5 Cancer Research Center (CRC), Shahid Beheshti University of Medical Sciences, Tehran, Iran; 6 Department of Paramedicine, Amol School of Paramedical Sciences, Mazandaran University of Medical Sciences, Sari, Iran; 7 Student Research Committee, Jahrom University of Medical Sciences, Jahrom, Iran; 8 Section of Environmental Health, Department of Public Health, University of Copenhagen, Copenhagen, Denmark; 9 Department of Environmental Medicine and Public Health, Icahn School of Medicine at Mount Sinai, New York, NY USA; 10 Department of Medical Education, Paul L. Foster School of Medicine, Texas Tech University Health Sciences Center El Paso, El Paso, TX USA; 11 Institute for Climate Change, Environmental Health and Exposomics, Icahn School of Medicine at Mount Sinai, New York, NY USA

**Keywords:** Breast cancer, Cancer epidemiology, Environmental sciences, Risk factors

## Abstract

This study aimed to examine the impacts of single and multiple air pollutants (AP) on the severity of breast cancer (BC). Data of 1148 diagnosed BC cases (2008–2016) were obtained from the Cancer Research Center and private oncologist offices in Tehran, Iran. Ambient PM_10_, SO_2_, NO, NO_2_, NO_X_, benzene, toluene, ethylbenzene, m-xylene, p-xylene, o-xylene, and BTEX data were obtained from previously developed land use regression models. Associations between pollutants and stage of BC were assessed by multinomial logistic regression models. An increase of 10 μg/m^3^ in ethylbenzene, o-xylene, m-xylene, and 10 ppb of NO corresponded to 10.41 (95% CI 1.32–82.41), 4.07 (1.46–11.33), 2.89 (1.08–7.73) and 1.08 (1.00–1.15) increase in the odds of stage I versus non-invasive BC, respectively. Benzene (OR, odds ratio = 1.16, 95% CI 1.01–1.33) and o-xylene (OR = 1.18, 1.02–1.38) were associated with increased odds of incidence of BC stages III & IV versus non-invasive stages. BC stage I and stage III&IV in women living in low SES areas was associated with significantly higher levels of benzene, ethylbenzene, o-xylene, and m-xylene. The highest multiple-air-pollutants quartile was associated with a higher odds of stage I BC (OR = 3.16) in patients under 50 years old. This study provides evidence that exposure to AP is associated with increased BC stage at diagnosis, especially under premenopause age.

## Introduction

Ambient air pollution (AAP) is a complex mixture of various gaseous pollutants and solid particles^1,2^. According to the World Health Organization (WHO) Ambient Air Quality Database 2022, more than 80% of populations living in urban areas with air monitoring devices, are exposed to air quality levels exceeding WHO thresholds^3^. AAP is estimated to have caused 4.2 million premature deaths globally in 2019^4^. Many studies indicate that AAP can increase the risk of myocardial infarction^5^, stroke^6^, headache^7–10^, disorders in fetal development^11^, asthma^12^, Chronic obstructive pulmonary disease (COPD)^2^, Attention deficit hyperactivity disorder (ADHD) in children^13^, and neurological diseases^14^. Furthermore, in 2013, AAP has been classified by the International Agency for Research on Cancer (IARC) as a Group 1 human carcinogen, mostly due to the evidence which related it to lung cancer^15^.

According to the report of the IARC in 2020, breast cancer (BC) has now become the most prevalent cancer worldwide, surpassing lung cancer^16^. It is also one of the leading causes of death globally^17,18^ and its burden has been increasing in many parts of the world over the past decades^19^. In 2020, 2.3 million women worldwide were diagnosed with BC, which corresponds to 1 in 8 diagnosed cancers; and 685,000 deaths occurred, which accounts for 1 in 6 deaths due to cancer in women^18,20,21^. By the end of 2020, there were 7.8 million women diagnosed with BC in the recent 5 years, making it the world's most common cancer^21^. It is predicted that by 2040, the burden of BC will increase to over 3 million morbidities and 1 million mortalities annually^19^.

BC is a complex multifactorial disease with a couple of known risk factors including female gender, older age, history of BC in the family, overweight and obesity, physical inactivity, history of radiation exposure, reproductive history (such as early menarche and late first pregnancy), tobacco and alcohol use, history of other benign breast diseases, short breastfeeding periods, and postmenopausal hormone therapy or oral contraceptives^21,22^. However, almost half of diagnosed breast cancers in women have no detectable risk factor^21^, suggesting a need to identify still unknown risk factors.

There is growing evidence that AAP can be a risk factor for BC^23,24^. Ecological studies propose that BC risk is higher in urban areas with higher air pollution compared to rural areas^25^. AAP contains many carcinogens that may perform as endocrine disruptors and cause oxidative DNA-damage which may affect BC risk^25–27^. However, studies have indicated inconsistent results about the impact of AAP on BC^23,24,28–30^. Uncertainty about the effect of AAP on BC is because it is difficult to prove causality due to the long latent period as well as low-dose exposure in the environment^31^. Meanwhile, the dissimilar findings of published studies can be partly justified by the diversity in AAP and exposure measurement methods and variations in study design^32^. Therefore, as Wei et al. 2021 recommended, there is a need to conduct studies especially in developing countries, with improved exposure measurement and covariate adjustments^33^.

In this regard, many researchers have explored a multiple-pollutant (instead of single pollutant) approach to evaluate the effects of air pollution, because humans are usually exposed to a complex mixture of air pollutants^34^, and in models that assess the impact of a single pollutant, it is difficult to determine whether an observed association reflects the impact of the specific pollutant being investigated, or the effect of other pollutants coinciding with it^35^. Although a few studies have shown the effect of air pollution on the incidence of BC^36^, but there is limited information about its effect on the severity of the disease. Therefore, the objective of this study was to investigate the effect of multiple air pollutants on BC stages diagnosed in Tehran, Iran. Also, as a secondary objective we assessed the pollutants’ impact on BC stages in different socio-economic levels.

## Methods

### Study subjects

Data was inquired from the Cancer Research Center (CRC) of Shahid Beheshti University of Medical Sciences in Tehran. All eligible female patients diagnosed with BC (ICD-O-3 C50.0–C50.9) according to pathology report, between 2008 and 2016 in different districts of Tehran were included. The Institutional Review Board (IRB) of the CRC approved the study protocol.

Data about patients’ characteristics including demographic factors (age at diagnosis, education level, and marital status), lifestyle factors (smoking status), reproductive factors (ages at first menstruation and pregnancy, number of pregnancies and deliveries), Estrogen/Progesterone receptor status, and clinical pathologic information including stage at diagnosis (non-aggressive, stage I, II, or III&IV), number of metastatic lymph nodes, family history of BC and diabetes was available for each patient. The frequency of missing data for all variables was low (≤ 5.5%).

Ethical approval was obtained (Code: IR.SBMU.CRC.REC.1400.008) from the Ethics Committee of the CRC of Shahid Beheshti University of Medical Sciences in Tehran, Iran, and all methods were performed under the relevant guidelines and regulations.

### Residence, highway proximity, and neighbourhood socioeconomic status

Residential addresses at diagnosis were geocoded to latitude and longitude coordinates using address or street locators.

An index of socioeconomic status (SES) was created based on principal component analysis of sixteen district -based indicators of SES. The socioeconomic indicators of the 22 districts of Tehran were extracted from a local study^37^. This SES index was assigned to participants’ addresses at diagnosis and was categorized into quartiles.

The distance of each patient to the highway was assigned as a proxy for traffic-related exposures. The distance of each patient’s address at diagnosis to the nearest main street or highway was calculated in the ESRI 2016 data layer. Distance to the highway was categorized in 3 groups as < 400, 400 to 800, and > 800 m.

### Long-term air pollution exposure assessment

Land use regression (LUR) models, were used to estimate exposure levels of PM_10_ (Particulate matter smaller than 10 microns), SO_2_ (sulphur dioxide), NO (nitric oxide), NO_2_ (nitrogen dioxide)_,_ and NO_X_ (oxides of nitrogen) based on measurements conducted at 23 regulatory network monitoring sites in Tehran, in 2010^38,39^. The volatile organic compound (VOC) concentration levels were obtained from spatial models that were built using long term measurements across about 180 sites in Tehran with very good performance^40^. More details about the exposure assessment methods have been described elsewhere^41^. Based on the patient’s geocoded residential locations at the time of diagnosis of breast cancer, air pollution exposure was estimated for each patient.

The ArcGIS Software (ArcGIS Locator version 10.0, ESRI, Redlands, CA, USA) and Tehran ArcGIS Shapefile Map Layers were used to geocode the residential addresses of the study subjects (X and Y coordinates of addresses).

### Statistical analyses

Data were summarized with mean ± standard deviation (SD) for continuous and frequency (percentage) for categorical variables. Chi-square tests was used to assess the difference among categorical variables in different categories of BC. Kolmogorov–Smirnov test was used to test the normality of the pollutants data and because the data were not normally distributed, Spearman’s correlation test was used to examine the correlations between different air pollutants and SES status.

The statistical analysis consisted of three steps: at first, we applied the weighted quantile sum (WQS) regression analysis to estimate the joint effect of air pollution mixtures on the stage of BC; in the second stage, we estimated the effect of each pollution and multipollutants on the stage of BC. Associations with BC risk were modeled using multinomial logistic regression models to estimate odds ratios (OR) and 95% confidence intervals (95% CI). Finally, the effect of each air pollutant on the stage of BC in different levels of SES was estimated.

The weighted quantile sum (WQS) regression analysis was done using the R package “gWQS” and Quantile-based g-Computation estimates was done suing the “qgcomp” package in R software. The WQS, developed specifically for the context of environmental mixtures analysis, is an increasingly common approach for multivariate regression in a high-dimensional dataset that operates in a supervised framework, creating a single score (the weighted quantile sum) that summarizes the overall exposure to the mixture, and by including this score in a regression model to evaluate the overall effect of the mixture on the outcome of interest^42^. The score is calculated as a weighted sum (so that exposures with weaker effects on the outcome have lower weight in the index) of all exposures categorized into quartiles (or more groups) so that extreme values have less impact on the weight estimation. A recent approach introduced by Keil et al. (2020) called Quantile-based g-Computation estimates the overall mixture effect with the same procedure used by WQS, but estimates the parameters of a marginal structural model, rather than the standard regression used in this study^43^. This approach, is under the common assumptions in causal inference such as exchangeability, causal consistency, positivity, no interference, and correct model specification. This model also improves the causal interpretation of the overall effect^44^.

After that, bivariate multinomial logistic regression analysis was conducted to explore the association of independent variables and BC stages. The cancer stage variable (outcome) had four categories: non-aggressive, stage I, stage II, stage III & IV, in which non-aggressive was the reference category. Thus, each pollutant and all confounder variables (with a *P*-value < 0.2 in bivariate analysis) were modeled by multivariate-adjusted multinomial logistic regression analysis. We also stratified the models by age (≥ 50 years old as menopause and < 50 years as pre-menopause).

Because the analysis examined the relations between BC stage and numerous correlated air pollutants, we used two different methods for parameterizing air pollutants in our study, single pollutant and multipollutant. The lowest-quantile category of multipollutants was used as the reference for comparison.

Finally, to understand differences in air pollutant and BC stage associations by SES, we assessed this association in low (quartile 1 and 2) and high (quartile 3 and 4) SES levels. Since data were from 22 districts, robust standard errors by cluster were incorporated into all analyses. Missing data were replaced by the variables’ mode or median value. Data description and analyses were conducted using STATA 17 and statistical software R (version 4.0.2, License GPLv2).

### Ethical approval and consent to participate

Participants in all studies provided written informed consent. Ethical approval was obtained (IR.SBMU.CRC.REC.1400.008) from the Ethics Committee of the Cancer Research Center (CRC) of Shahid Beheshti University of Medical Sciences, Tehran, Iran. The patient data was anonymous and strictly confidential.

## Results

### Study population characteristic

The study population consisted of 1164 BC cases aged 20 years and older residing in 22 urban districts of Tehran during 2008–2016. We had to exclude the data of 16 subjects who lived in remote suburbs of Tehran, which air pollutant surveillance was not done. Finally, 1148 cases entered the analyses.

**Figure 1:**
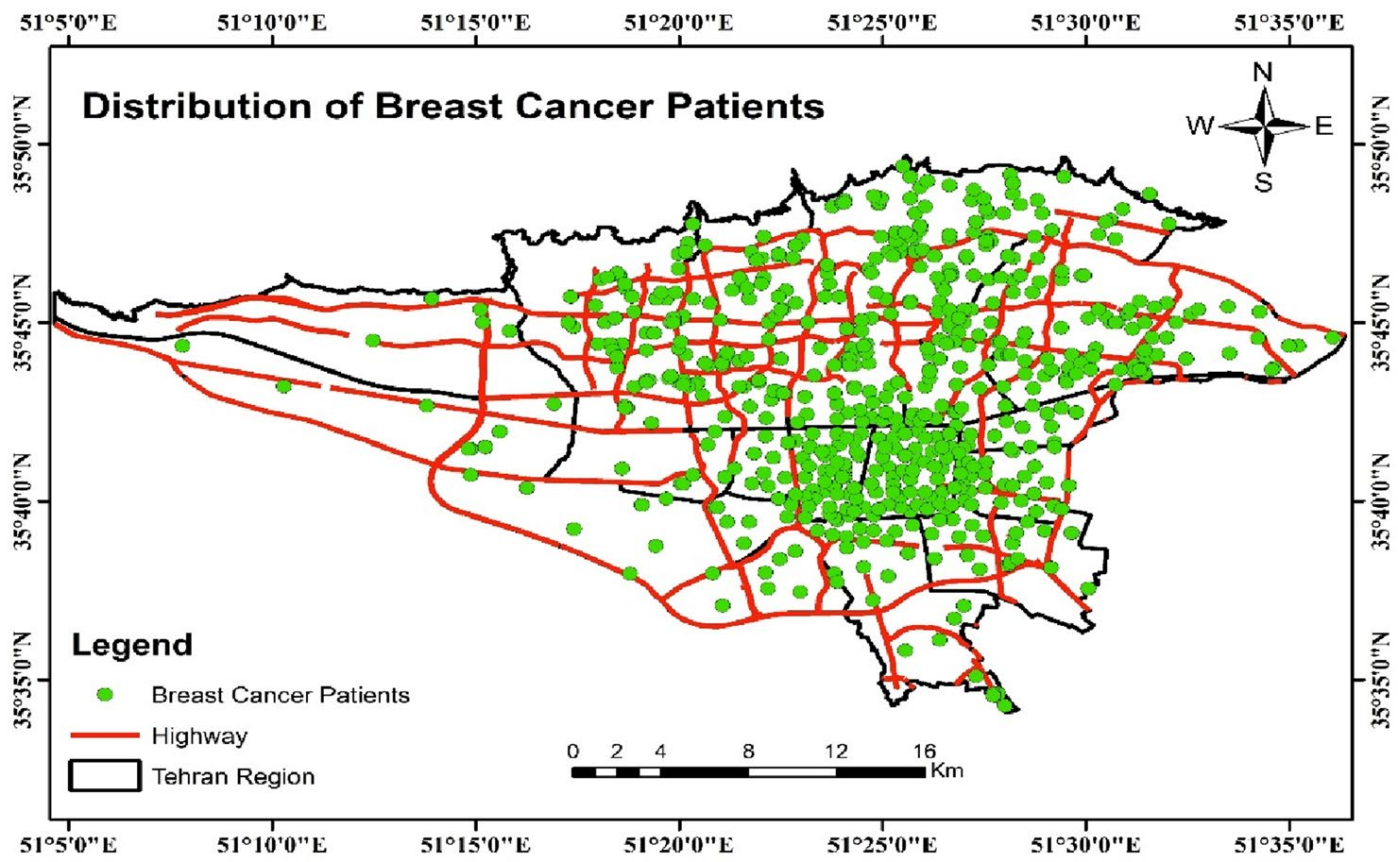
Spatial distribution of breast cancer patients in different areas of Tehran in 2008–2016 (n = 1148).

**Figure 2:**
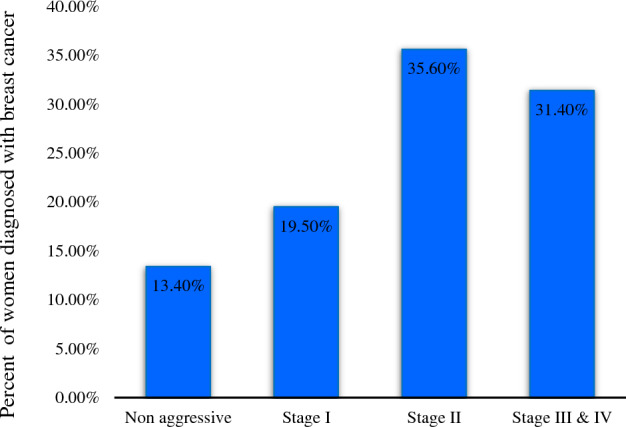
Breast cancer severity status among women diagnosed with breast cancer (n = 1148).

The distribution of BC patients in different regions of Tehran is shown in Fig. 1. The mean (standard deviation) of age at diagnosis in our study was 50.25 (11.67) years. Regarding the demographic and clinical variables, there were statistically significant differences in age at diagnosis, education level, marital status, family history of BC, diabetes, smoking, pregnancy status, and ER–PR status among patients diagnosed at different stages of BC (Table 1). The distribution of BC patients in different categories is shown in Table 1. The most common stage of BC was stage 2 with 35.6%, followed by stage III & IV with 31.40% of cases (Fig. 2).

**Table 1:** The demographic and clinical characteristics of women diagnosed with breast cancer in different areas of Tehran in 2008–2016.

Variables	Total, N (%) (n = 1148)	Non-aggressive (n = 154)	Stage I (n = 224)	Stage II (n = 409)	Stage III & IV (n = 361)	*P*-value
Age at diagnosis, years						
< 50	633 (55.1)	100 (15.8)	115 (18.2)	206 (32.5)	212 (33.5)	0.005
≥ 50	515 (44.9)	54 (10.5)	109 (21.2)	203 (39.4)	149 (28.9)	
Education level						
Academic	338 (29.8)	40 (11.8)	77 (22.8)	117 (34.6)	104 (30.8)	0.003
Diploma	354 (31.2)	39 (11.0)	78 (22.0)	134 (37.9)	103 (29.1)	
Under diploma	298 (26.3)	40 (13.4)	41 (13.8)	111 (37.2)	106 (35.6)	
Unknown	144 (12.7)	33 (22.9)	22 (15.3)	45 (31.2)	44 (30.6)	
Marital status						
Single	75 (6.6)	10 (13.3)	17 (22.7)	22 (29.3)	26 (34.7)	*P* < 0.001
Married	880 (76.9)	96 (10.9)	178 (20.2)	329 (37.4)	277 (31.5)	
Divorced/ Widowed	62 (5.4)	3 (4.8)	11 (17.7)	29 (46.8)	19 (30.6)	
Unknown	127 (11.1)	42 (33.1)	18 (14.2)	28 (22.0)	39 (30.7)	
Family history of BC						
No	674 (59.6)	63 (9.3)	131 (19.4)	267 (39.6)	213 (31.6)	*P* < 0.001
Yes	279 (24.7)	27 (9.7)	66 (23.7)	98 (35.1)	88 (31.5)	
Unknown	177 (15.7)	52 (29.4)	25 (14.1)	43 (24.3)	57 (32.2)	
Smoking status						
No	765 (66.6)	71 (9.3)	164 (21.4)	296 (38.7)	234 (30.6)	*P* < 0.001
Yes	58 (5.1)	7 (12.1)	8 (13.8)	23 (39.7)	20 (34.5)	
Secondhand smoke	85 (7.4)	6 (7.1)	17 (20.0)	24 (28.2)	38 (44.7)	
Unknown	240 (20.9)	70 (29.2)	35 (14.6)	66 (27.5)	69 (28.8)	
Diabetes						
No	490 (45.2)	41 (8.4)	101 (20.6)	193 (39.4)	155 (31.6)	*P* < 0.001
Yes	73 (6.7)	2 (2.7)	8 (11.0)	34 (46.6)	29 (39.7)	
Unknown	522 (48.1)	89 (17.0)	106 (20.3)	163 (31.2)	164 (31.4)	
Age at first menstruation, year						
≤ 12	767 (66.8)	110 (14.3)	152 (19.8)	263 (34.3)	242 (31.6)	0.430
> 12	381 (33.2)	44 (11.5)	72 (18.9)	146 (38.3)	119 (31.2)	
Age at first pregnancy, year						
None	267 (23.3)	60 (22.5)	44 (16.5)	70 (26.2)	93 (34.8)	*P* < 0.001
< 30	806 (70.2)	87 (10.8)	168 (20.8)	308 (38.2)	243 (30.1)	
≥ 30	75 (6.5)	7 (9.3)	12 (16.0)	31 (41.3)	25 (33.3)	
Number of pregnancies						
None	267 (23.3)	60 (22.5)	44 (16.5)	70 (26.2)	93 (34.8)	*P* < 0.001
1, 2	321 (28)	32 (10.0)	77 (24.0)	118 (36.8)	94 (29.3)	
≥ 3	560 (48.8)	62 (11.1)	103 (18.4)	221 (39.5)	174 (31.1)	
Number of deliveries						
None	280 (24.4)	62 (22.1)	45 (16.1)	76 (27.1)	97 (34.6)	*P* < 0.001
1, 2	458 (39.9)	48 (10.5)	107 (23.4)	172 (37.6)	131 (28.6)	
≥ 3	410 (35.7)	44 (10.7)	72 (17.6)	161 (39.3)	133 (32.4)	
Estrogen/Progesterone receptor status (ERPR status)						
ER + PR +	577 (63.6)	23 (4)	136 (23.6)	236 (40.9)	182 (31.5)	0.647
ER + PR-	87 (9.6)	4 (4.6)	18 (20.7)	30 (45.5)	35 (40.2)	
ER−PR +	37 (4.1)	1 (2.7)	9 (24.3)	13 (35.1)	14 (37.8)	
ER−PR-	206 (22.7)	5 (2.4)	39 (18.9)	92 (44.7)	70 (7.7)	
Number of metastatic lymph node						
None	672 (58.5)	148 (22.0)	219 (32.6)	232 (34.5)	73 (10.9)	*P* < 0.001
< 5	319 (27.8)	4 (1.3)	4 (1.3)	171 (53.6)	140 (43.9)	
≥ 5	157 (13.7)	2 (1.3)	1 (0.6)	6 (3.8)	148 (94.3)	
Socioeconomic status						
Quartile 1-low	277 (24.2%)	32 (11.6%)	60 (21.7%)	99 (35.7%)	86 (31%)	0.335
Quartile 2	308 (26.9%)	44 (14.3%)	52 (16.9%)	109 (35.4%)	103 (33.4%)	
Quartile 3	341 (29.8%)	47 (13.8%)	67 (19.6%)	135 (39.6%)	92 (27)	
Quartile 4- high	219 (19.1%)	29 (13.2%)	45 (20.5%)	66 (30.1%)	79 (36.1%)	

*BC* breast cancer, *PR* Progesterone receptor, *ER* Estrogen receptor.

Numbers may not total to 100% due to missing data.

### Generalized weighted quantile sum regression analysis (gWQS model)

**Figure 3:**
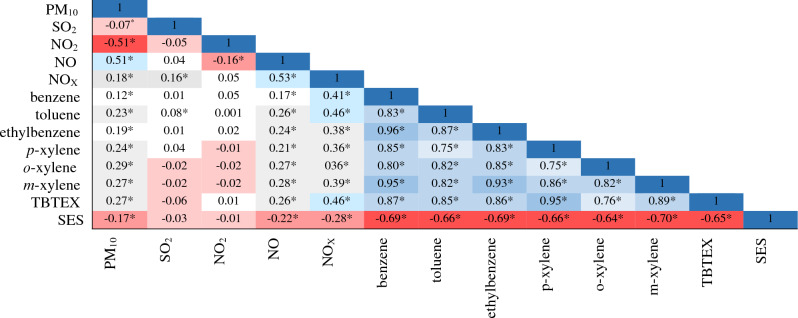
Spearman coefficients of correlation between air pollutants and SES in Tehran, Iran, during 2008–2016. SES: Socioeconomic Status, * Correlation is significant at P<0.01.

The Spearman correlations among pollutants and SES status is shown in Fig. 3. The highest values were among benzene compounds (benzene, toluene, ethylbenzene, m-xylene, p-xylene, o-xylene, and TBTEX) (*P* < 0.05). The SES status had inverse correlations with all pollutants varying from − 0.01 to − 0.70 (Fig. 3).

**Figure 4:**
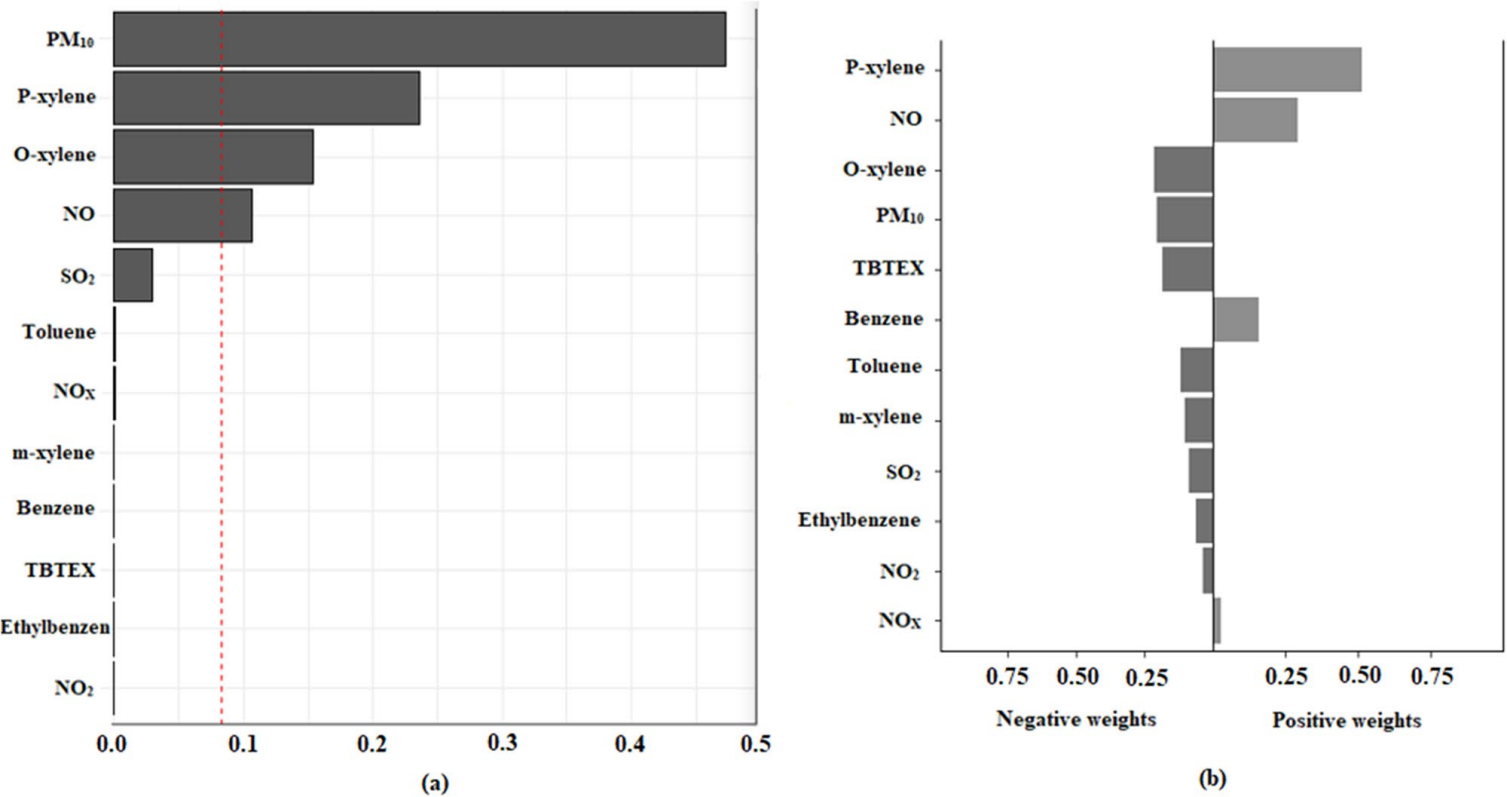
Distribution of (**a**) quantile sum regression model index weights and (**b**) positive and negative weights estimation in the simulated dataset of multipollutant percentile quartile for each air pollution.

Figure 4a shows the contribution of each pollutant to the construction of the composite variable (multi-pollution) used in the multipollutant model. In this figure, the red dotted line indicates the significance level, and according to the concentration and pollutant weights, the contributions of ethylbenzene, NO_2_, and benzene were relatively small, while the contribution of PM_10_, p-xylene, o-xylene, and NO were relatively prominent in the multipollution variable. Furthermore, Fig. 4b shows the positive and negative weight of variables in the construction of the multipollution variable. Also, of the four important and influential variables, p-xylene and NO variables had positive weights and o-xylene and PM_10_ variables had negative weights.

Summary statistics for each pollutant, multipollution variable, and proximity to the highway of BC cases are shown in Table 2.

**Table 2:** Summary of air pollution and highway proximity variables.

Variables	Mean	SD	Min	Max	IQR	25th	50th	75th
Single pollution								
PM_10_ (μg/m^3^)	96.10	3.74	3.14	184.00	42.13	74.96	101.35	117.09
SO_2_ (ppb)	58.84	3.79	8.35	158.00	47.53	33.39	54.71	80.92
NO_2_ (ppb)	69.18	2.31	13.00	124.00	14.83	58.11	63.62	72.94
NO (ppb)	91.50	4.98	7.36	327.26	76.67	50.32	82.42	126.99
NO_X_ (ppb)	2.06	1.08	54.00	523.01	135.11	124.48	182.2	259.59
Benzene (μg/m^3^)	8.62	2.51	3.12	23.68	3.13	6.68	8.41	9.81
Toluene (μg/m^3^)	25.29	7.47	7.84	98.88	10.62	19.14	24.79	29.76
Ethylbenzene (μg/m^3^)	6.10	1.72	2.31	17.00	2.3	4.69	5.94	6.99
P-xylene(μg/m^3^)	5.91	1.71	1.76	28.69	1.89	4.73	5.69	6.62
O-xylene(μg/m^3^)	6.28	1.79	2.80	14.42	2.44	4.79	6.01	7.23
m-xylene(μg/m^3^)	11.12	3.58	3.86	31.98	4.52	8.45	10.59	12.97
TBTEX (μg/m^3^)	62.99	2.02	25.22	321.37	24.81	47.33	62.25	72.14
Multipollutant variable	42.20	18.94	0.44	83.73	26.01	30.67	43.68	56.68
Highway proximity (m)	6.58	5.68	1.38	3639.44	5.58	2.69	5.71	8.27

*SD* standard deviation, *Min* Minimum, *Max* Maximum, *IQR* Interquartile range.

### Severity of BC and ambient air pollutants analysis

Table 3 shows the Odds ratios (OR) and 95% confidence intervals (95% CI) of the crude analyses of the associations between the independent variables and cancer stages by multinomial logistic regression analyses. Education level, smoking status, diabetes, family history of BC, age at first menstruation, number of pregnancies and deliveries, and highway proximity were included in the multivariate model, because, at least one of their categories had a *P*-value < 0.2.

**Table 3:** Crude Odds ratio (OR) between independent variables and breast cancer stages.

Variables	< 50 year (n = 633)			≥ 50 year (n = 515)		
	Stage I (n = 115)	Stage II (n = 206)	Stages III & IV (n = 212)	Stage I (n = 109)	Stage II (n = 203)	Stages III & IV (n = 149)
Education level (Ref: Academic)						
Diploma	1.28 (0.64–2.56)	1.37 (0.72–2.62)	1.35 (0.71–2.58)	0.67 (0.27–1.68)	0.80 (0.34–1.89)	0.62 (0.24–1.57)
Under diploma	**0.26** (0.10–0.63)*	0.66 (0.34–1.31)	0.58 (0.29–1.16)	0.61 (0.24–1.54)	0.94 (0.39–2.21)	1.50 (0.62–3.66)
Marital status (Ref = single)						
Married	1.21 (0.49–2.93)	1.74 (0.75–4.06)	1.57 (0.69–3.59)	†	†	†
Divorced/ Widowed	1.15 (0.16–8.27)	2.81 (0.50–15.77)	1.39 (0.23–8.51)	†	†	†
Smoking status (Ref = No)						
Yes	0.25 (0.04–1.42)	0.69 (0.20–2.36)	0.64 (0.18–2.35)	0.70 (0.16–3.01)	0.81 (0.22–3.01)	1.10 (0.29–4.21)
Secondhand smoke	1.00 (0.32–3.10)	0.68 (0.22–2.06)	1.29 (0.46–3.62)	2.46 (0.29–20.95)	2.25 (0.28–17.96)	4.98 (0.63–39.06)
Diabetes (Ref = No)						
Yes	1.34 (0.14–14.04)	2.45 (0.30–20.03)	1.01 (0.11–9.42)	1.67 (0.18–15.42)	4.13 (0.52–32.93)	7.21 (0.90–57.71)
Family history of BC(Ref = No)						
Yes	**1.76** (0.85–3.65)*	1.09 (0.55–2.17)	1.02 (0.51–2.05)	0.69 (0.30–1.56)	0.59 (0.28–1.28)	0.85 (0.39–1.85)
Age at first menstruation, year (Ref = ≤ 12)						
> 12	1.13 (0.62–2.06)	1.52 (0.89–2.56)	1.36 (0.81–2.30)	1.16 (0.58–2.34)	1.19 (0.63–2.27)	1.03 (0.53–2.02)
Age at first pregnancy, year (Ref: < 30)						
≥ 30	1 (0.27–3.71)	0.62 (0.17–2.19)	0.78 (0.22–2.69)	0.76 (0.18–3.21)	1.70 (0.48–5.99)	1.79 (0.49–6.52)
Number of pregnancies (Ref: No)						
1 and 2	**2.58** (1.29–5.13)*	**2.92** (1.58–5.39)*	**2.12** (1.17–3.83)*	**5.46** (1.90–15.71)*	**4.07** (1.48–11.19)*	1.10 (0.36–3.32)
≥ 3	**2** (1.04–3.86)*	**2.21** (1.24–3.96)*	1.27 (0.72–2.25)	**2.29** (1.02–5.13)*	**3.65** (1.76–7.56)*	**2.42** (1.18–4.96)*
Number of deliveries (Ref: No)						
1 and 2	**2.59** (1.37–4.87)*	**2.58** (1.47–4.51)*	**1.89** (1.10–3.26)*	**4.13** (1.68–10.11)*	**3.77** (1.63–8.71)*	1.31 (0.54–3.16)
≥ 3	1.96 (0.94–4.07)	**1.99** (1.05–3.80)*	1.12 (0.58–2.14)	2.23 (0.97–5.13)	**3.65** (1.73–7.72)*	**2.79** (1.33–5.88)*
Estrogen/Progesterone receptor status (ERPR status) (Ref: ER−PR-)						
ER + PR +	1.02 (0.34–3.02)	0.78 (0.27–2.25)	0.73 (0.25–2.09)	†	†	†
ER + PR-	0.56 (0.08–3.82)	0.68 (0.12–3.89)	1.06 (0.19–5.95)	†	†	†
ER−PR +	0.91 (0.08–9.98)	0.31 (0.03–3.59)	0.64 (0.06–6.42)	†	†	†
Number of metastatic lymph nodes (Ref: No)						
< 5	2 (0.11–35.81)	**19.33** (2.30–160.2)*	0.68 (0.11–4.19)	0.49 (0.03–8.07)	†	†
≥ 5	2.34 (0.21–26.17)	0.81 (0.13–4.97)	**0.01** (0.00–0.05)*	†	†	†
Air pollution						
PM_10_ (μg/m^3^)**	1.04 (0.97–1.12)	1.01 (0.94–1.07)	1.03 (0.96–1.09)	.98 (0.89–1.07)	1.00 (0.92–1.08)	1.01 (0.93–1.10)
SO_2_ (ppb)	0.99 (0.92–1.06)	1.00 (0.94–1.07)	0.98 (0.93–1.05)	0.93 (0.86–1.02)	0.97 (0.90–1.05)	**0.91** (0.84–0.99)*
NO_2_ (ppb)	1.08 (0.97–1.21)	1.03 (0.93–1.14)	1.02 (0.93–1.14)	0.98 (0.85–1.13)	0.97 (0.85–1.11)	0.95 (0.83–1.09)
NO (ppb)	**1.06** (1.01–1.12)*	1.04 (0.99–1.09)	1.03 (0.98–1.08)	0.96 (0.89–1.08)	1.00 (0.94–1.07)	1.01 (0.94–1.07)
NO_X_ (ppb)	1.02 (0.99–1.05)	1.00 (0.98–1.03)	0.99 (0.97–1.02)	0.98 (0.95–1.02)	0.99 (0.97–1.02)	0.98 (0.96–1.02)
Benzene (μg/m^3^)	**3.78** (1.22–11.78)*	2.04 (0.72–5.80)	0.97 (0.33–2.82)	0.69 (0.19–2.52)	1.27 (0.40–4.05)	1.43 (0.44–4.71)
Toluene (μg/m^3^)	**1.54** (1.05–2.24)*	1.25 (0.88–1.76)	1.13 (0.80–1.59)	0.88 (0.56–1.39)	1.16 (0.77–1.74)	1.22 (0.81–1.58)
Ethylbenzene (μg/m^3^)	**9.98** (1.85–58.82)*	3.18 (0.67–15.03)	1.57 (0.33–7.51)	0.48 (0.07–3.21)	1.41 (0.26–7.62)	2.08 (0.36–11.75)
P-xylene(μg/m^3^)	6.61 (0.89–48.55)	2.14 (0.34–13.44)	1.36 (0.22–8.63)	0.44 (0.05–3.53)	1.93 (0.35–10.75)	1.79 (0.31–10.49)
O-xylene(μg/m^3^)	4.21 (0.94–18.89)	1.63 (0.41–6.55)	1.31 (0.33–5.27)	0.63 (0.09–4.19)	1.44 (0.26–7.99)	2.77 (0.48–16.16)
m-xylene(μg/m^3^)	**2.55** (1.15–5.67)*	1.52 (0.72–3.19)	1.09 (0.52–2.31)	0.83 (0.33–2.04)	1.28 (0.57–2.90)	1.29 (0.56–2.99)
TBTEX (μg/m^3^)	**1.24** (1.05–1.46)*	1.09 (0.94–1.27)	1.07 (0.92–1.24)	0.95 (0.79–1.13)	1.06 (0.92–1.23)	1.04 (0.89–1.22)
Multipollutant level (Ref: Quartile 1-low)						
Quartile 2	0.77 (0.37–1.63)	0.81 (0.43–1.53)	1.02 (0.54–1.92)	1.02 (0.42–2.05)	0.92 (0.41–2.05)	0.98 (0.42–2.27)
Quartile 3	1.03 (0.49–2.15)	0.78 (0.40–1.48)	0.95 (0.49–1.81)	1.48 (0.58–3.78)	1.51 (0.63–3.57)	1.82 (0.75–4.44)
Quartile 4-high	**2.75** (1.18–6.39)*	**2.29** (1.06–4.95) *	2.07 (0.95–4.52)	1.05 (0.43–2.57)	0.90 (0.39–2.05)	0.99 (0.42–2.34)
Highway proximity (Ref: > 800 m)						
< 400 m	1.03 (0.51–2.08)	1.27 (0.67–2.41)	0.78 (0.41–1.47)	0.94 (0.39–2.24)	1.08 (0.49–2.38)	1.68 (0.74–3.82)
400–800 m	0.81 (0.41–1.58)	1.14 (0.62–2.09)	0.86 (0.47–1.55)	1.12 (0.51–2.49)	0.88 (0.42–1.83)	1.26 (0.52–2.45)

*BC* breast cancer, *PR* Progesterone receptor, *ER* Estrogen receptor.

*P < 0.05.

**The Odds ratio is estimated for each 10 unit increase in pollutants.

^†^The numbers in the subgroups were too low to calculate the OR.

An increase of 10 μg/m^3^ in ethylbenzene, o-xylene, m-xylene, and 10 ppb of NO in the adjusted model corresponded to OR = 10.41 (95% CI 1.32–82.41), 4.07 (95% CI 1.46–11.33), 2.89 (95% CI 1.08–7.73) and 1.08 (95% CI 1.00–1.15) increases in the odds of stage I BC in comparison to non-invasive BC, respectively. Also, benzene (OR = 1.16, 95% CI 1.01–1.33) and o-xylene (OR = 1.18, 95% CI 1.02–1.38) were significantly associated with an increased odds of BC (stages III & IV) compared to non-aggressive stages. However, an increase of 10 ppb in SO_2_ was significantly associated with a decreased odds of stage III and IV BC, in patients over 50 years old (Table 4).

**Table 4:** Adjusted Odds ratios and 95% confidence intervals between each 10-unit increase in air pollutants and breast cancer stages.

Air pollution	< 50 year (n = 633)			≥ 50 year (n = 515)		
Single pollution	Stage I (n = 115)	Stage II (n = 206)	Stages III & IV (n = 212)	Stage I (n = 109)	Stage II (n = 203)	Stages III & IV (n = 149)
PM_10_ (μg/m^3^)	1.06 (0.97–1.16)	1.01 (0.93–1.09)	1.07 (0.97–1.17)	0.96 (0.86–1.07)	1.02 (0.92–1.13)	1.06 (0.94–1.18)
SO_2_ (ppb)	0.96 (0.88–1.06)	1.04 (0.96–1.14)	1.07 (0.96–1.18)	0.9 (0.81–1.00)	0.98 (0.88–1.08)	**0.87** (0.77–0.98)*
NO_2_ (ppb)	1.07 (0.93–1.23)	1.02 (0.89–1.17)	1.01 (0.86–1.17)	1.00 (0.83–1.20)	0.98 (0.82–1.17)	0.96 (0.78–1.18)
NO (ppb)	**1.08** (1.00–1.15)*	1.05 (0.98–1.12)	1.04 (0.96–1.12)	0.98 (0.90–1.07)	1.03 (0.95–1.11)	1.02 (0.93–1.12)
NO_X_ (ppb)	1.01 (0.98–1.05)	1.00 (0.97–1.03)	0.99 (0.96–1.02)	0.98 (0.94–1.01)	0.99 (0.96–1.03)	0.99 (0.95–1.03)
Benzene (μg/m^3^)	3.71 (0.92–15.04)	1.76 (0.45–6.86)	1.18 (0.25–5.58)	0.44 (0.10–1.87)	1.32 (0.34–5.06)	**1.16** (1.01–1.33)*
Toluene (μg/m^3^)	1.44 (0.89–2.32)	1.17 (0.74–1.84)	1.17 (0.70–1.95)	0.66 (0.38–1.14)	1.00 (0.59–1.67)	0.99 (0.55–1.82)
Ethylbenzene (μg/m^3^)	**10.41** (1.32–82.41)**	2.63 (0.35–19.47)	2.12 (0.22–20.48)	0.20 (0.02–2.01)	1.15 (0.13–9.86)	1.58 (0.13–19.51)
P-xylene(μg/m^3^)	9.01 (0.72–111.94)	1.93 (0.16–22.76)	1.80 (0.11–28.48)	0.19 (0.02–1.76)	1.69 (0.31–9.26)	1.84 (0.24–14.28)
O-xylene(μg/m^3^)	**4.07** (1.46–11.33)**	1.44 (0.23–8.80)	1.58 (0.21–12.24)	0.23 (0.03–2.04)	1.24 (0.16–9.61)	**1.18** (1.02–1.38)*
m-xylene(μg/m^3^)	**2.89** (1.08–7.73)*	1.48 (0.57–3.89)	1.35 (0.45–4.02)	0.59 (0.21–1.68)	1.36 (0.52–3.56)	1.44 (0.46–4.52)
TBTEX (μg/m^3^)	1.18 (0.97–1.45)	1.04 (0.86–1.27)	1.04 (0.83–1.29)	0.88 (0.73–1.07)	1.05 (0.90–1.22)	1.04 (0.86–1.25)
Multipollutant level (Ref: Quartile 1-low)						
Quartile 2	0.96 (0.39–2.35)	0.84 (0.38–1.82)	1.22 (0.56–2.61)	1.05 (0.37–2.97)	0.89 (0.34–2.32)	0.94 (0.35–2.54)
Quartile 3	1.15 (0.47–2.78)	0.83 (0.38–1.82)	1.09 (0.51–2.38)	1.33 (0.48–3.69)	1.21 (0.47–3.12)	1.42 (0.54–3.75)
Quartile 4-high	**3.16** (1.17–8.53)**	**2.16** (0.87–5.31)*	2.14 (0.86–5.32)	1.07 (0.39–2.94)	0.96 (0.37–2.46)	1.07 (0.46–2.48)

Reference group: non-aggressive stage of breast cancer.

Adjusted for education level, smoking status, diabetes, family history of BC, age at first menstruation, number of pregnancies, and highway proximity.

*P < 0.05, **P < 0.01.

In patients under 50 years old, in multi-pollutant models, the high multiple-air-pollution quartile was associated with higher odds of stage I BC (OR = 3.16, 95% CI 1.17–8.53) when compared with the low multiple-air-pollution quartile (Table 4).

Furthermore, our results showed that the adjusted odds of BC stage I and stage III&IV, and air pollution exposure was higher among low SES cases (stages III & IV vs non-aggressive: OR_O-xylene_ = 2.69 and OR_M-xylene_ = 1.83, stages I vs non-aggressive: OR_Benzene_ = 3.67, OR_Ethylbenzene_ = 7.15, and OR_O-xylene_ = 2.49) (Table 5).

**Table 5:** Adjusted Odds ratios and 95% confidence intervals of the association between each 10 unit increase in air pollutants and stage of cancers in SES categories.

Air pollution	SES level	Stages of BC		
		Stage I (n = 224)	Stage II (n = 409)	Stages III & IV (n = 361)
PM_10_ (μg/m^3^)	Low SES(Q1-Q2)	0.99 (0.91–1.07)	1.02 (0.95–1.11)	1.04 (0.96–1.12)
	High SES(Q3-Q4)	1.03 (0.96–1.11)	0.99 (0.92–1.06)	1.01 (0.94–1.08)
SO_2_ (ppb)	Low	0.91 (0.83–0.99)	0.99 (0.91–1.06)	0.96 (0.89–1.04)
	High	1.02 (0.95–1.09)	1.02 (0.96–1.08)	0.96 (0.89–1.03)
NO_2_ (ppb)	Low	1.04 (0.91–1.19)	1.03 (0.91–1.16)	0.97 (0.86–1.09)
	High	1.05 (0.93–1.18)	0.99 (0.89–1.12)	1.02 (0.91–1.15)
NO (ppb)	Low	1.01 (0.95–1.08)	1.02 (0.96–1.08)	1.03 (0.98–1.09)
	High	1.03 (0.97–1.09)	1.03 (0.97–1.08)	1.01 (0.95–1.07)
NO_X_ (ppb)	Low	1.01 (0.99–1.04)	1.01 (0.98–1.04)	1.01 (0.98–1.04)
	High	1.00 (0.97–1.03)	0.99 (0.97–1.02)	0.99 (0.95–1.01)
Benzene (μg/m^3^)	Low	**3.67** (1.04–10.87)*	2.45 (0.82–7.31)	1.95 (0.65–5.86)
	High	0.52 (0.06–4.33)	1.55 (0.23–10.68)	0.23 (0.03–1.63)
Toluene (μg/m^3^)	Low	1.22 (0.82–1.80)	1.37 (0.90–2.10)	1.40 (0.95–2.07)
	High	1.03 (0.58–1.83)	1.46 (0.87–2.45)	0.87 (0.51–1.49)
Ethylbenzene (μg/m^3^)	Low	**7.15** (1.14–44.98)*	2.96 (0.54–16.16)	3.57 (0.65–19.60)
	High	0.81 (0.05–14.34)	3.39 (0.25–45.65)	0.37 (0.03–5.28)
P-xylene (μg/m^3^)	Low	3.43 (0.46–25.34)	3.73 (0.55–25.29)	3.31 (0.48–22.85)
	High	0.13 (0.00–4.39)	1.37 (0.05–35.14)	0.16 (0.01–4.31)
O-xylene (μg/m^3^)	Low	**2.49** (1.31–4.74)*	1.77 (0.36–8.62)	**2.69** (1.27–5.74)*
	High	0.52 (0.05–5.49)	1.29 (0.16–10.47)	0.39 (0.04–3.54)
M-xylene (μg/m^3^)	Low	2.25 (0.97–5.22)	1.81 (0.83–3.98)	1**.83** (0.99–3.39)*
	High	0.82 (0.19–3.40)	1.37 (0.38–4.96)	0.52 (0.14–1.96)
TBTEX (μg/m^3^)	Low	1.13 (0.96–1.34)	1.14 (0.97–1.34)	1.13 (0.96–1.32)
	High	1.05 (0.80–1.38)	1.13 (0.88–1.44)	0.94 (0.73–1.21)

Reference group: non-aggressive stage of breast cancer.

Adjusted for education level, smoking status, diabetes, family history of BC, number of pregnancies, and highway proximity.

*BC* breast cancer, *SES* socioeconomic status.

*P ≤ 0.05.

## Discussion

To our best knowledge, this is the first study to examine the association between air pollutants (single and mixtures) and BC severity. The findings suggest that air pollutant exposure, especially in patients who were diagnosed under 50 years old was associated with a higher stage of BC at diagnosis. This might mean that air pollutants are increasing the speed of breast cancer development and progress.

Similar to various other studies^45^, the spatial correlations between the individual air pollutants were rather high (− 0.51 to 0.96); however our study used a new approach and investigated the effect of a mixture of air pollutants as well. The interest in determining the simultaneous effect of multiple pollutant exposure on health outcomes, and the identification of dominant pollutants has been growing in recent years. These studies can probably explain the health outcomes much better than single pollutant studies^45–48^.

A recent approach introduced by Keil et al. (2020) called Quantile-based g-Computation gWQS estimates the overall mixture effect with the same procedure used by WQS, but estimates the parameters of a marginal structural model, rather than the standard regression. This model also improves the causal interpretation of the overall effect of multiple pollutants^40^. It combines pollutants into a weighted additive index, which is used to estimate an overall mixture effect through a bootstrap resampling procedure and avoids overfitting and collinearity^42^. This model has been used in an increasing number of studies^42,49,50^. Our study indicated that the dominant pollutants in the gWQS model were p-xylene, NO, o-xylene, and PM_10_.

Our results showed a significant association between air pollution and BC severity by adjusting for smoking status, diabetes, family history of BC, age at first menstruation, number of pregnancies, and highway proximity. Our study demonstrated some associations between ethylbenzene, o-xylene, m-xylene, and NO and stage of BC among women under 50 years old and o-xylene and benzene among women over 50 years old in univariate models. Previous studies also suggest air pollution might be related to breast cancer, particularly among women with a positive family history and age of under 50 years old^32,51^.

One meta-analysis of 36 effect estimates for PM_2.5_, PM_10_, and NO_2_ has confirmed that decreasing long-term NO_2_ exposure or correlated air pollutant exposures could lower breast cancer risk; and also showed that associations of NO_2_ levels with breast cancer risk were higher in premenopausal than in postmenopausal women^52^. In this current study, a significant association was seen between air pollutant exposures and severity of BC in premenopausal women. This shows that the effect of air pollutants on BC can be different in different periods of a women’s life and may be stronger during premenopause. Differences in cancer morphology or hormonal subtypes in pre- and post-menopausal women might explain this difference in the effect of air pollutants^53^. Some studies found positive associations between air pollution and BC in postmenopausal women^54,55^.

Two recent reviews suggested an significant increased risk of breast cancer associated with an increase in nitrogen dioxide (NO_2_) and nitrogen oxide (NOx) levels, both of which are proxies for traffic exposure^56,57^. Also a nested case–control study within the French E3N cohort showed an increased odds of breast cancer associated with long-term exposure to NO_2_ air pollution^54^.

Hwang et al. in a nationwide analysis in South Korea (2005–2016) showed that the ambient air pollutant concentrations were positively and signifcantly associated with breast cancer odds, and per 10 ppb NO_2_ increase, the odds of BC increased by OR = 1.14 (95% CI = 1.12–1.16)^58^. A cohort study conducted between 1980 and 1985 in urban centers in Canada, showed that exposure to NO_2_ increases the risk of premenopausal breast cancer, and the rate ratio (RR) for an increase of 9.7 ppb (the interquartile range) was 1.13 (95% CI 0.94–1.37) among premenopausal patients^59^.

Studies have shown that NO can directly inhibit the activity of caspases providing an efficient means to block apoptosis and can increase breast cancer development through estrogen and progesterone pathways, which are both involved in the carcinogenesis of breast cancer^60^.

Results of a review study in 2018 showed that many individual air pollutants are genotoxic and some are estrogenic or anti-estrogenic. The polycyclic aromatic hydrocarbons (PAHs) are the most-studied component of air pollution in relation to breast cancer and include hundreds of compounds and their metabolites with different biologic activities which are thought to specifically caused mammary gland tumors^36^. PAHs also activate CYP3A4 via PXR receptors, and can affect estrogen metabolism through these routes as well^61^. The role of PAHs in tumor progression, has been suggested in some studies^62^, and the results from other studies done in different geographic locations showed that some VOCs are human carcinogens with strong evidence for genotoxicity, increased PAH-DNA adducts, TP53 polymorphisms and mutations^36^.

Although a nationwide analysis in South Korea showed that SO_2_ concentrations were positively and significantly associated with the odds of breast cancer (per 1 ppb SO_2_, OR = 1.04, 95% CI = 1.02–1.05)^58^, we found inverse effects of SO_2_ on the severity of breast cancer among women over 50 years old.

In this study, we found negative and statistically significant correlations between air pollution and SES level. Interestingly, significant associations were seen between air pollution and BC severity in low SES regions as well. The high correlation between SES and air pollutants suggests that part of the effect of air pollutants on BC maybe explained by low SES. Population features, neighborhood deprivation, and air pollution levels are often interconnected, although the direction of associations maybe different in different areas^63^. Recent studies suggest this pattern could be linked to the composition of the air pollution mixture or the intrinsic vulnerability of the population^52^. A recent Multiethnic Cohort (MEC) among African American, European American, Japanese American, and Latina American women diagnosed with breast cancer reported the harmful impact of air pollutants on breast cancer survival, and that this association may be confounded by socioeconomic factors^64^. One American study reported that the worst socio-demographic environmental quality, increased the odds of distant metastatic breast cancer by 10% in non-metro-urbanized counties (OR 1.10; 95% CI 1.00–1.20, *P* = 0.035)^65^.

In this study, breast cancer patients data was collected from the Shahid Beheshti University of Medical Sciences Cancer Research Center and oncologists private offices, which included patients from all areas of Tehran. But it did not cover all the patients in Tehran.

Up to our knowledge, this is the largest study to date to examine the association between air pollutants and BC in Iran and the first study to explore the associations between BC severity and single and multiple air pollutants.

This study has the advantage of using WQS inference and the flexible g-computation method which allows to explore the nonlinear and non-additive effects of individual pollutants and their mixture as a whole. Quantile g-computation is able to estimate the parameters of a marginal structural model^43^. In the grouped weighted quantile sum (GWQS), multiple groups of pollutants are allowed to be included in the GWQS regression model, and the components of the multi-pollutant mixture are allowed to have different magnitudes and directions^42^.

In this study confounders were controlled for in the analysis; however, additional information on some potentially important individual confounders, such as genetic predisposition of patients for certain cancer types, diet, physical activity, and exposure to indoor pollutants were not available, and this could have led to residual confounding.

Another limitation of this study was that we used exposure data gathered at a single point in time analyzed with LUR models to estimate the concentration of air pollutants in the long run. Nevetheless, the temporal stability of these models for traffic-related air pollution has been shown in studies. Researchers have commented that LUR models were able to provide reliable estimates for a period of 7 years in Vancouver^66^.

We had no information about the residential history of patients which could have confounded our analyses. Additional studies are needed to determine the effect of relocation on air pollution exposure and the incidence and severity of BC.

Another limitation of this study was that we tested multiple hypothesis with a type I error equal to 0.05, and some of these comparison might have become significant by chance.

## Conclusion

In summary, we found substantial evidence that higher air pollutants particularly NO, ethylbenzene, o-xylene, m-xylene, and benzene in outdoor air were associated with increased odds of BC stages at diagnosis. Furthermore, the association between air pollutants and BC severity appeared higher in premenopausal women. Our work has implications for future environmental justice studies investigating the influence of SES on the association between air pollutants and BC. Additionally, more research on this association will improve our understanding of the mechanisms underlying the role of air pollutants on the severity of BC.

## Data Availability

The datasets generated during and/or analysed during the current study are available from the corresponding author on reasonable request. To meet ethical requirements for the use of confidential patient data, requests must be approved by the Ethical committee of the Cancer Research Center (CRC) of Shahid Beheshti University of Medical Sciences, Tehran, Iran.

## Abbreviations

BC: Breast cancer
AAP: Ambient air pollution
WHO: World Health Organization
COPD: Chronic obstructive pulmonary disease
ADHD: Attention deficit hyperactivity disorder
IARC: International Agency for Research on Cancer
BMI: Body mass index
CRC: Cancer Research Center
IRB: Institutional Review Board
LUR: Land use regression
VOC: Volatile organic compound
WQS: Weighted quantile sum
SO_2_: Sulphur dioxide
NO: Nitric oxide
NO_2_: Nitrogen dioxide
NOx: Oxides of nitrogen
PM_10_: Particulate matter smaller than 10 microns
